# A rare presentation of Sheehan’s syndrome with recurrent hypoglycemia: A case report

**DOI:** 10.1097/MD.0000000000049008

**Published:** 2026-05-22

**Authors:** Niraj Bam, Milan Pokhrel, Bibek Shrestha, Grishma Kandel, Sonam Dhenga

**Affiliations:** a Department of Pulmonology and Critical Care Medicine, Tribhuvan University Teaching Hospital, Kathmandu, 44600, Nepal.

**Keywords:** adrenal insufficiency, hypopituitarism, postpartum hemorrhage, sheehan syndrome

## Abstract

**Rationale:**

Sheehan syndrome is a rare but potentially fatal cause of hypopituitarism resulting from ischemic necrosis of the anterior pituitary following severe postpartum hemorrhage. Because symptoms often develop gradually and nonspecifically, diagnosis is frequently delayed until acute illness precipitates adrenal crisis or severe hypoglycemia.

**Patient concerns:**

A 48-year-old woman presented with central chest pain, productive cough, dyspnea, intermittent fever, severe anemia, and recurrent hypoglycemia. She had a history of severe postpartum hemorrhage 20 years earlier, followed by secondary amenorrhea and agalactia.

**Diagnoses:**

Physical examination revealed basal crepitations. Laboratory investigations demonstrated microcytic hypochromic anemia, leukocytosis with neutrophilia, hyponatremia, markedly low morning cortisol (0.6 µg/dL), low FT3 and FT4, and low-normal TSH, consistent with panhypopituitarism. Based on the obstetric history and hormonal profile, a diagnosis of Sheehan syndrome was established.

**Interventions:**

The patient was treated with glucocorticoid replacement therapy, followed by levothyroxine supplementation. Supportive management for the respiratory tract infection and hypoglycemia was also provided.

**Outcomes:**

The patient showed significant clinical improvement after initiation of hormonal replacement therapy, with stabilization of blood glucose levels and improvement in systemic symptoms.

**Lessons:**

This case highlights the prolonged latency between postpartum hemorrhage and presentation of Sheehan syndrome, with acute infection acting as a trigger for adrenal insufficiency. Clinicians should maintain high suspicion for Sheehan syndrome in women with a history of complicated delivery, lactation failure, amenorrhea, and chronic endocrine symptoms, even decades after the inciting event. Early recognition and prompt hormone replacement are lifesaving.

## 1. Introduction

Sheehan syndrome is a rare but fatal complication of severe postpartum hemorrhage, which results from ischemia and necrosis of anterior pituitary gland.^[1]^ It often leads to varying degrees of hypopituitarism, which follow insidious onset after the inciting event.^[2]^ The clinical manifestations can be vague and nonspecific, including secondary amenorrhea, fatigue, cold intolerance, and loss of secondary sexual characteristics, leading to underdiagnosis or delayed diagnosis of the illness.^[3]^ Acute presentations, particularly those precipitated by infections, can unmask the symptoms due to hormonal deficiencies, with complications including adrenal crisis and severe hypoglycemia.^[4]^

We report a case of a 48-year-old woman with a past history of postpartum hemorrhage (PPH) who presented to our hospital with the symptoms of acute respiratory tract infection, severe anemia, and recurrent hypoglycemia. Further evaluation revealed panhypopituitarism consistent with Sheehan syndrome. This case highlights the importance of maintaining a high index of suspicion for hypopituitarism in women with a history of complicated deliveries such as PPH, even decades after the obstetric event, and underscores the need for timely hormonal evaluation to prevent serious morbidity. This case report has been reported following CARE guideline.^[5]^

## 2. Case presentation

A 48-year-old female, a nonsmoker and nonalcohol consumer, presented to the emergency department with multiple complaints. Her primary concern was central chest pain persisting for the past 10 days. The pain was described as a constant, dull ache, exacerbated by deep inspiration, and occasionally radiating anteriorly. She also reported generalized body weakness that had progressively worsened over the past week. The patient described a history of intermittent cough over the same duration, which was occasionally productive of yellowish sputum. The cough episodes were not associated with hemoptysis or wheezing but had shortness of breath of Modified Medical Research Council (mMRC) grade III to IV. For the past 2 days, she experienced fever on and off, with a maximum recorded temperature of 102°F, unaccompanied by chills or rigor. There were no associated night sweats or weight loss reported. The patient had a significant obstetric history: during the delivery of her second child approximately 20 years ago, she experienced severe postpartum hemorrhage requiring prolonged hospitalization. Following that event, she noted complete absence of menstruation (secondary amenorrhea) and an inability to lactate despite a live birth. She did not seek medical attention at that time due to limited healthcare access and assumed these changes were part of normal postpartum recovery. Over the years, she experienced persistent fatigue, cold intolerance, and gradual thinning of scalp and body hair, which were never evaluated by a healthcare professional. She denied any history of hypertension, diabetes mellitus, tuberculosis, thyroid disease, or cardiac illness. There was no family history of endocrine disorders, autoimmune conditions, or malignancy. She was not on any regular medications and had no known drug allergies.

On general examination, the patient was conscious, alert, and well oriented to time, place, and person. She appeared well built and nourished, with no evidence of pallor, icterus, cyanosis, clubbing, lymphadenopathy, or pedal edema. At the time of assessment, she was afebrile, with a pulse rate of 78 beats per minute and regular rhythm, blood pressure of 118/76 mm Hg, respiratory rate of 16 breaths per minute, and oxygen saturation of 98% on room air. Systemic examination revealed no abnormalities. The respiratory system showed bilateral vesicular breath sounds of reduced intensity and Basal crepitations present. Cardiovascular examination demonstrated normal first and second heart sounds without any murmurs, rubs, or gallops. On ophthalmic evaluation fundus examination revealed normal optic disc and macula in both eyes, with no evidence of papilledema or visual field defects.

The patient’s blood investigation revealed a hemoglobin level of 6.9 g/dL and a packed cell volume of 21.5%, indicating severe anemia. Given the severity of her anemia (Hb 6.9 g/dL), the patient received packed red cell transfusion upon admission, which resulted in stabilization of hemoglobin levels. The total leukocyte count was elevated at 14,500/cumm, with a neutrophil predominance of 70%, suggestive of leukocytosis with neutrophilia, likely reflecting an underlying infectious process. He also had recurrent hypoglycemia episodes with glucose level maintained between 48–56 mg/dL. The packed cell volume of 26.4% and a reduced red blood cell count of 3.63 million/cumm, consistent with anemia. Red cell indices showed a low mean corpuscular volume of 72.6 fL, mean corpuscular hemoglobin of 21.3 pg, and mean corpuscular hemoglobin concentration of 29.3%, indicating a microcytic hypochromic pattern. The red cell distribution width was elevated at 16.5%. Platelet count was within normal limits at 215,000/cumm. Peripheral smear examination demonstrated hypochromic, microcytic red blood cells with anisopoikilocytosis, along with the presence of target cells and tear drop cells. The urea level of 2.3 mmol/L was noted, which is within the normal range, and a creatinine level of 174 μmol/L, elevated above the reference range. Serum sodium was mildly reduced at 134 mEq/L, while potassium was within normal limits at 3.6 mEq/L.

Hormonal profile revealed markedly low morning serum cortisol at 0.6 μg/dL (reference: 3.7–19.4 μg/dL), confirming adrenal insufficiency. Thyroid function tests showed low free triiodothyronine (<1.64 pmol/L; reference: 2.4–6.0 pmol/L) and free thyroxine (<5.15 pmol/L; reference: 10.0–19.0 pmol/L) with low-normal TSH (0.956 μIU/mL; reference: 0.35–4.94 μIU/mL), consistent with central hypothyroidism. Gonadotropin profile demonstrated suppressed luteinizing hormone (1.06 mIU/mL; reference: 5.1–26.5 mIU/mL) and low follicle-stimulating hormone (5.28 mIU/mL; reference for postmenopausal: 21.5–131.0 mIU/mL). Serum prolactin was within the low-normal range at 3.83 ng/mL (reference: 3.5–5.2 ng/mL). Growth hormone was undetectable (<0.05 ng/mL; reference: <8.0 ng/mL). These findings, in the context of her history, were suggestive of panhypopituitarism. Adrenocorticotropic hormone (ACTH), Dehydroepiandrosterone sulfate (DHEAS), and estradiol assays were not available at our center at the time of evaluation; however, the existing hormonal profile was sufficient to establish panhypopituitarism.

**Figure 1.**
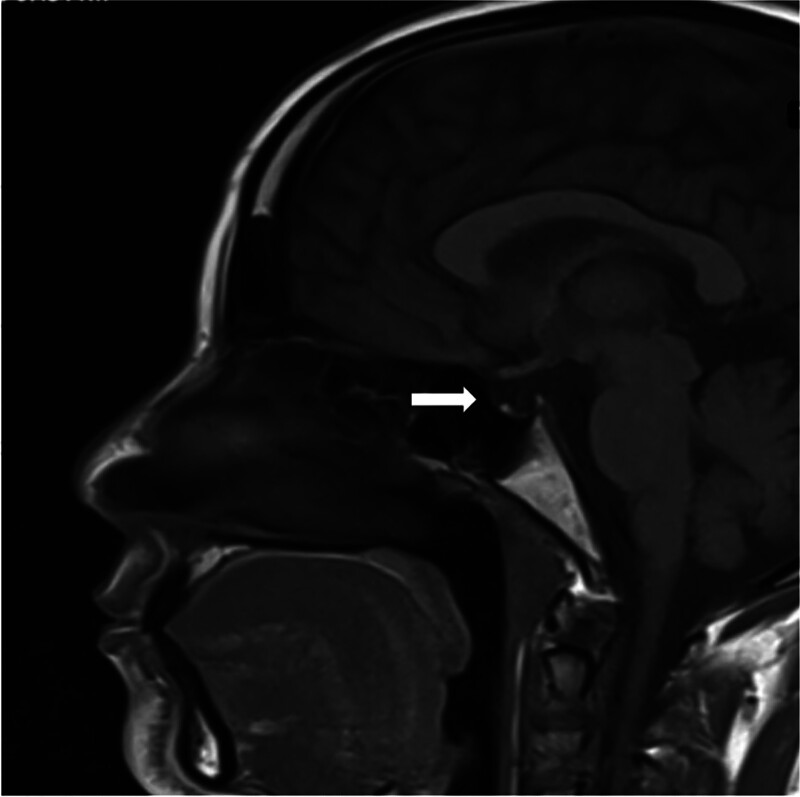
Sagittal T1W MRI image shows the sella is empty, i.e pituitary gland appears shrunken, and CSF fills the space instead (shown by white arrow).

**Figure 2.**
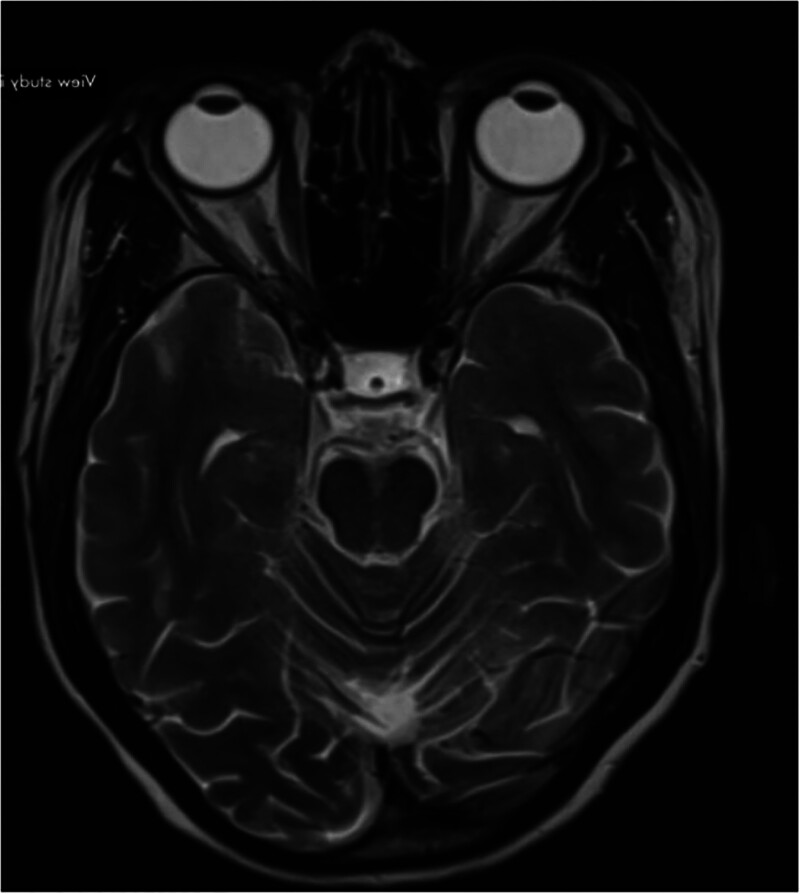
Axial T2W MRI image shows empty sella with a small T2 dot of the infundibulum (shown by white arrow).

Magnetic resonance imaging (MRI) of the brain (Figure 1, Figure 2) demonstrated a completely empty sella, with the pituitary gland not visualized and the sella filled with cerebrospinal fluid. A posterior pituitary bright spot was noted on T1-weighted images and T2 weighted images. The size and configuration of the sella turcica were within normal limits, with intact floor and walls. The infundibulum was midline and of normal size. The optic chiasm, suprasellar cisterns, cavernous sinuses, and visualized internal carotid arteries were unremarkable. Ventricular system and basal cisterns were normal in size and configuration, with no evidence of mass effect, midline shift, edema, hemorrhage, or infarction. Posterior cranial fossa, brainstem, craniocervical junction, and paranasal sinuses appeared normal.

Based on the patient’s history of severe postpartum hemorrhage, subsequent secondary amenorrhea and agalactia, clinical features of chronic hormone deficiency, biochemical evidence of panhypopituitarism (low cortisol, central hypothyroidism, low gonadotropins, undetectable growth hormone), recurrent hypoglycemia, and MRI findings of an empty sella with preserved posterior pituitary bright spot, the diagnosis was: Sheehan’s syndrome presenting with panhypopituitarism and recurrent hypoglycemia, precipitated by respiratory tract infection. The patient was started on oral glucocorticoid replacement with prednisolone 10 mg daily to address adrenal insufficiency, with plans for lifelong maintenance and stress-dose adjustments during periods of illness or surgery. After 2–3 days of corticosteroid therapy, levothyroxine was initiated to correct central hypothyroidism, following the recommended sequence to avoid precipitating adrenal crisis. Hypoglycemia was managed with intravenous glucose infusion. A structured summary of the patient’s hormonal and laboratory profile is presented in Table to provide clear chronological interpretation of the biochemical abnormalities. (Table 1 and Table 2)

**Table 1:** Laboratory investigation of the patient.

Parameter	Patient value	Reference range	Interpretation
Morning Cortisol	0.6 μg/dL	3.7–19.4 μg/dL	Very low – adrenal insufficiency
Free T3 (FT3)	<1.64 pmol/L	2.4–6.0 pmol/L	Low – central hypothyroidism
Free T4 (FT4)	<5.15 pmol/L	10–19 pmol/L	Low – central hypothyroidism
TSH	0.956 μIU/mL	0.35–4.94 μIU/mL	Inappropriately normal – central hypothyroidism
LH	1.06 mIU/mL	5.1–26.5 mIU/mL	Low – hypogonadism
FSH	5.28 mIU/mL	21.5–131 mIU/mL (postmenopausal)	Low – hypogonadism
Prolactin	3.83 ng/mL	3.5–5.2 ng/mL	Low-normal – supports hypopituitarism
Growth Hormone	<0.05 ng/mL	<8.0 ng/mL	Very low – GH deficiency
Serum Sodium	134 mEq/L	135–145 mEq/L	Mild hyponatremia
Glucose (recurrent)	48–56 mg/dL	70–140 mg/dL	Recurrent hypoglycemia
Hemoglobin	6.9 g/dL	12–16 g/dL	Severe microcytic anemia
WBC Count	14,500/cumm (Neutrophils 70%)	4000–11,000	Leukocytosis with neutrophilia

**Table 2:** Chronological clinical timeline.

Time period	Events
20 years ago	Severe postpartum hemorrhage → Secondary amenorrhea, agalactia
Years after PPH	Chronic fatigue, cold intolerance, hair thinning (never evaluated)
10 days before admission	Chest pain, dyspnea, cough
2 days before admission	Fever, worsening weakness
At presentation	Severe anemia, recurrent hypoglycemia, infection
During evaluation	Hormonal panel → Panhypopituitarism
After starting steroids	Stabilization of glucose and symptoms
After levothyroxine	Clinical improvement

## 3. Discussion

Sheehan syndrome is a rare but serious cause of hypopituitarism, resulting from ischemic necrosis of the anterior pituitary gland secondary to severe PPH.^[1,2]^ The mean interval between PPH and diagnosis of SS is reported to range from 7 to 20 years in various studies, especially in resource-limited settings where obstetric and endocrine services are less accessible.^[6]^ The prolonged lag period between postpartum hemorrhage and eventual diagnosis of Sheehan’s syndrome in our patient is consistent with reports from similar resource-limited settings. Boro et al described a 50-year-old woman who presented 27 years after severe PPH with spontaneous conception and pseudohypertrophic myopathy, demonstrating that delays exceeding 2 decades are not uncommon in low-resource environments where postpartum endocrine follow-up is limited.^[7]^ This delay is clinically significant because chronic hormone deficiencies predispose patients to acute decompensation during periods of physiological stress, as occurred in our case with an acute respiratory tract infection precipitating adrenal crisis and severe hypoglycemia.^[8]^

The classical pathophysiology of Sheehan syndrome is attributed to ischemic necrosis of the anterior pituitary following severe postpartum hemorrhage and hypovolemic shock. However, accumulating evidence suggests that autoimmunity may contribute to the progressive pituitary dysfunction observed in some patients. Studies have demonstrated the presence of antipituitary and antihypothalamic antibodies years after the obstetric insult, implying that ischemia may initiate an autoimmune cascade that perpetuates glandular destruction.^[9]^ More recent data also indicate that lymphocytic infiltration and autoimmune hypophysitis may coexist in a subset of Sheehan syndrome cases, potentially explaining delayed or evolving hormonal deficits even decades after PPH.^[10]^ This dual mechanism – initial ischemic injury followed by chronic autoimmune-mediated damage – may account for the wide variation in clinical severity and the prolonged interval between PPH and presentation.

Hypoglycemia in sheehan syndrome is multifactorial, primarily resulting from cortisol deficiency and, to a lesser extent, hypothyroidism.^[11]^ In our patient, low morning cortisol (0.6 μg/dL), reduced FT3 and FT4 with inappropriately normal-low TSH, and chronic amenorrhea were diagnostic of panhypopituitarism. The concurrent presentation with severe anemia, recurrent hypoglycemia, and chest infection made the initial presentation misleading. Such nonspecific symptoms may lead to misdiagnosis such as sepsis, pneumonia, or primary cardiac/respiratory disease.

Common symptoms include agalactia, amenorrhea, hypothyroidism, and asthenia. Diagnosis can be challenging due to the nonspecific nature of symptoms and the long interval since the obstetric event. Early signs such as agalactia and amenorrhea are often overlooked, contributing to the diagnostic delay [6]. The most affected pituitary hormones are growth hormone and prolactin, followed by gonadotropins, thyroid-stimulating hormone, and adrenocorticotropic hormone.^[9]^ Key diagnostic clues include a history of postpartum hemorrhage, lactation failure, and amenorrhea.^[12]^ Acute illnesses, such as dengue fever or influenza, can precipitate adrenal crises in patients with undiagnosed Sheehan syndrome.^[13]^ Recurrent hypoglycemia is a less recognized but potentially life-threatening manifestation of this condition.^[14]^ Respiratory tract infections, as in this case, can amplify metabolic stress, increase glucose utilization, and further deplete already low cortisol reserves.^[13]^ The patient’s recurrent hypoglycemia episodes (48–56 mg/dL) were a direct manifestation of this endocrine vulnerability. Importantly, corticosteroid replacement prior to initiating levothyroxine was correctly prioritized to avoid precipitating adrenal crisis, aligning with established management protocols. Radiological investigations play a crucial role in diagnosis. MRI provides detailed findings, showing a partially empty sella and pituitary atrophy.^[15]^ The sella turcica volume is significantly lower in Sheehan syndrome patients compared to healthy individuals.^[16]^

Treatment involves hormone replacement therapy, with glucocorticoids and levothyroxine being crucial. Hematological abnormalities, such as anemia, leucopenia, and thrombocytopenia, are common in Sheehans’ syndrome patients but can be reversed with appropriate hormone replacement.^[17]^ Our patient improved clinically after corticosteroid and levothyroxine supplementation, underscoring the reversibility of acute manifestations with timely intervention.Several reports emphasize that Sheehan syndrome is still prevalent in developing countries, where obstetric hemorrhage remains a major cause of maternal morbidity.^[18]^

Compared with previously published cases of Sheehan’s syndrome, our patient demonstrates several notable differences that expand current understanding of the condition. Most reported cases describe presentations dominated by chronic hypothyroid or adrenal insufficiency symptoms, whereas only a minority highlight recurrent hypoglycemia as the leading manifestation.^[2]^ Moreover, hypoglycemia in the literature is often precipitated by severe infections such as dengue fever, influenza, or gastrointestinal illness; however, reports of respiratory tract infections triggering metabolic decompensation are sparse.^[4]^ The typical interval between postpartum hemorrhage and diagnosis ranges from 7 to 20 years, consistent with our patient, but the combination of severe anemia, recurrent hypoglycemia, and respiratory illness causing acute unmasking is rarely documented.^[19]^ Importantly, unlike most published cases, our patient exhibited profound hypoglycemia despite only mild hyponatremia and no signs of hemodynamic collapse, suggesting a subtler but clinically significant endocrine vulnerability. These distinctions highlight a key knowledge gap in existing literature namely, the limited characterization of hypoglycemia-dominant presentations of Sheehan’s syndrome and the underreporting of respiratory infections as precipitating factors. Our case adds to the evidence that metabolic instability may precede overt adrenal crisis and emphasizes the need for high clinical suspicion even in atypical presentations.

This case underscores several important clinical lessons. First, clinicians should maintain a high index of suspicion for hypopituitarism in any woman with a history of severe postpartum hemorrhage followed by secondary amenorrhea and agalactia, regardless of how many years have passed since delivery. Second, intercurrent illnesses such as respiratory infections can precipitate adrenal crises in undiagnosed Sheehan syndrome, manifesting as life-threatening hypoglycemia and hemodynamic instability. Third, the correct therapeutic sequence is crucial – glucocorticoid replacement should always precede thyroid hormone initiation in suspected or confirmed adrenal insufficiency to avoid precipitating adrenal crisis.

## 4. Conclusion

This case emphasizes that Sheehan’s syndrome can remain clinically silent for decades and may present atypically with recurrent hypoglycemia triggered by common infections such as respiratory illness. Clinicians should actively inquire about past obstetric complications – especially postpartum hemorrhage – in any woman presenting with unexplained hypoglycemia, hyponatremia, or chronic constitutional symptoms. Early morning cortisol and thyroid function testing should be prioritized in these patients, as timely identification of adrenal insufficiency and initiation of glucocorticoids can prevent life-threatening metabolic decompensation. This report highlights the need for heightened awareness in resource-limited settings, routine postpartum endocrine follow-up for high-risk mothers, and standardized screening protocols to reduce the persistent delay in diagnosing Sheehan’s syndrome.

## Author contributions

**Conceptualization:** Niraj Bam, Milan Pokhrel, Bibek Shrestha.

**Writing – original draft:** Niraj Bam, Milan Pokhrel, Bibek Shrestha, Grishma Kandel, Sonam Dhenga.

**Writing – review & editing:** Niraj Bam, Milan Pokhrel, Bibek Shrestha, Grishma Kandel, Sonam Dhenga.
